# A review of the genus *Gaurenoglaea* Ronkay, Ronkay, Gyulai & Hacker, 2010 (Lepidoptera, Noctuidae) with description of a new species from Xizang Autonomous Region, China

**DOI:** 10.3897/BDJ.12.e141076

**Published:** 2024-12-02

**Authors:** Liang Guo, Qian Yan, Min Wang, Wa Da

**Affiliations:** 1 Department of Entomology, College of Plant Protection, South China Agricultural University, Guangzhou, China Department of Entomology, College of Plant Protection, South China Agricultural University Guangzhou China; 2 Institute of International Rivers and Eco-Security, Yunnan University, Kunming, China Institute of International Rivers and Eco-Security, Yunnan University Kunming China; 3 Tibet Plateau Institute of Biology, Lhasa, China Tibet Plateau Institute of Biology Lhasa China; 4 Motuo Biodiversity Observation and Research Station of Xizang Autonomous Region, Linzhi, China Motuo Biodiversity Observation and Research Station of Xizang Autonomous Region Linzhi China

**Keywords:** *Gaurenoglaea*, Xylenini, Medog, Tibet, Himalayan Region, taxonomy

## Abstract

**Background:**

*Gaurenoglaea* is a genus belonging to the tribe Xylenini. Since its establishment more than ten years ago, this genus has included only two species.

**New information:**

A new species of the genus *Gaurenoglaea* Ronkay, Ronkay, Gyulai & Hacker, 2010, *G.medogensis* **sp. nov.** is described and illustrated from Xizang Autonomous Region, south-western China. The new species can be distinguished from the previous *Gaurenoglaea* species by the differences of forewing cilia and aedeagus. A key to the *Gaurenoglaea* species, based on morphology, is provided.

## Introduction

The genus *Gaurenoglaea* was established by Ronkay et al. (2010b) with two closely-related species: *Gaurenoglaeamisconspicua* Ronkay, Ronkay, Gyulai & Hacker, 2010 and *Gaurenoglaeaalternata* Ronkay, Ronkay, Gyulai & Hacker, 2010. The former, collected from north-western Vietnam, was designated as the type species and the latter was collected from south-western China. The two *Gaurenoglaea* species have a similar appearance, making it difficult to distinguish them by external features. The ground colour of the upper side of their forewings is dark brown, dotted with many ochreous light patches of different sizes, making the pattern unique within the tribe Xylenini. More intriguingly, their common appearance is similar to that of the Orthosiini species *Houlberthosiaornatissima* (Wileman, 1911). If only the external features are examined, we will intuitively, yet mistakenly, think that they are inextricably linked to the genus *Houlberthosia* (Wileman 1911, Ronkay et al. 2010a). According to Ronkay et al. (2010b), this genus is closely related to *Hemiglaea* Sugi, 1980, *Rhynchaglaea* Hampson, 1906 and *Owadaglaea* Hacker & Ronkay, 1997. Although this interesting genus was established over a decade ago, no new *Gaurenoglaea* species have been discovered since.

Motuo (= Medog) is a beautiful county situated in the southeast of the Xizang Autonomous Region (= Tibet), featuring the renowned Yarlung Zangbo Grand Canyon. Unlike other extensive areas in Xizang, the summer monsoon from the Indian Ocean brings a humid climate here. Thanks to numerous advantageous conditions, a wealth of unique species have been found to thrive in this habitat. However, in the past, due to the difficult transportation circumstances, many places in this area had never been explored by humans, impeding the discovery of new species. With the completion of the Motuo Highway, these unfavourable conditions have been significantly improved. Close to the tenth anniversary of its completion, we have finally discovered the third species of *Gaurenoglaea* in Motuo County, which will be described in this paper.

## Materials and methods

Specimens for this study are held in the following collections: South China Agricultural University (SCAU), Hungarian Natural History Museum (HNHM, Budapest, Hungary), coll. Gábor Ronkay (GRB, Budapest, Hungary), coll. Péter Gyulai (PGM, Miskolc, Hungary).

The specimens of the new species were photographed using a Nikon CoolPix S7000 digital camera. The abdomens were removed and macerated in a hot 10% sodium hydroxide (NaOH) solution for the examination of genitalia. Photos of the genitalia were taken under a Carl Zeiss Discovery V12 digital microscope. Photos of the habitat were taken with a Nikon D7100 digital camera or a mobile phone model ELS-AN10. All of the photos were processed by Adobe Photoshop CS5® software.

## Taxon treatments

### Gaurenoglaea

Ronkay, Ronkay, Gyulai & Hacker, 2010

B4E0E9E9-26FC-5CB7-9664-56B6D2E39267

Gaurenoglaea misconspicua Ronkay, Ronkay, Gyulai & Hacker, 2010Ronkay et al. 2010b: p. 280; fig. 65; pl. 53, fig. 2.

#### Diagnosis

**Figure 2.**
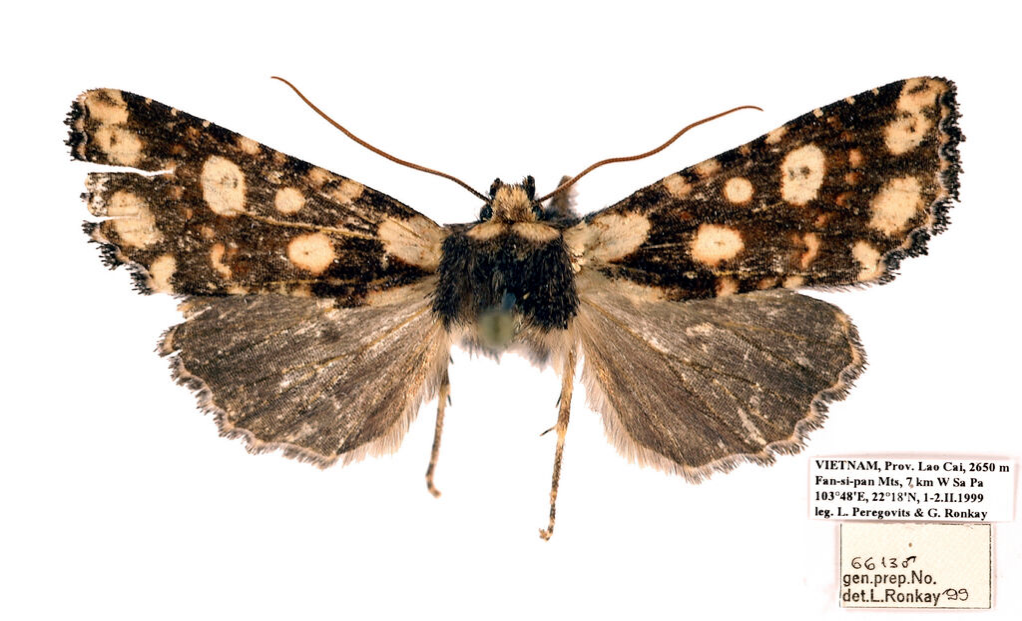
Adults and labels of *Gaurenoglaeamisconspicua*, male, holotype, Lao Cai, Vietnam (coll. GRB).

**Figure 5.**
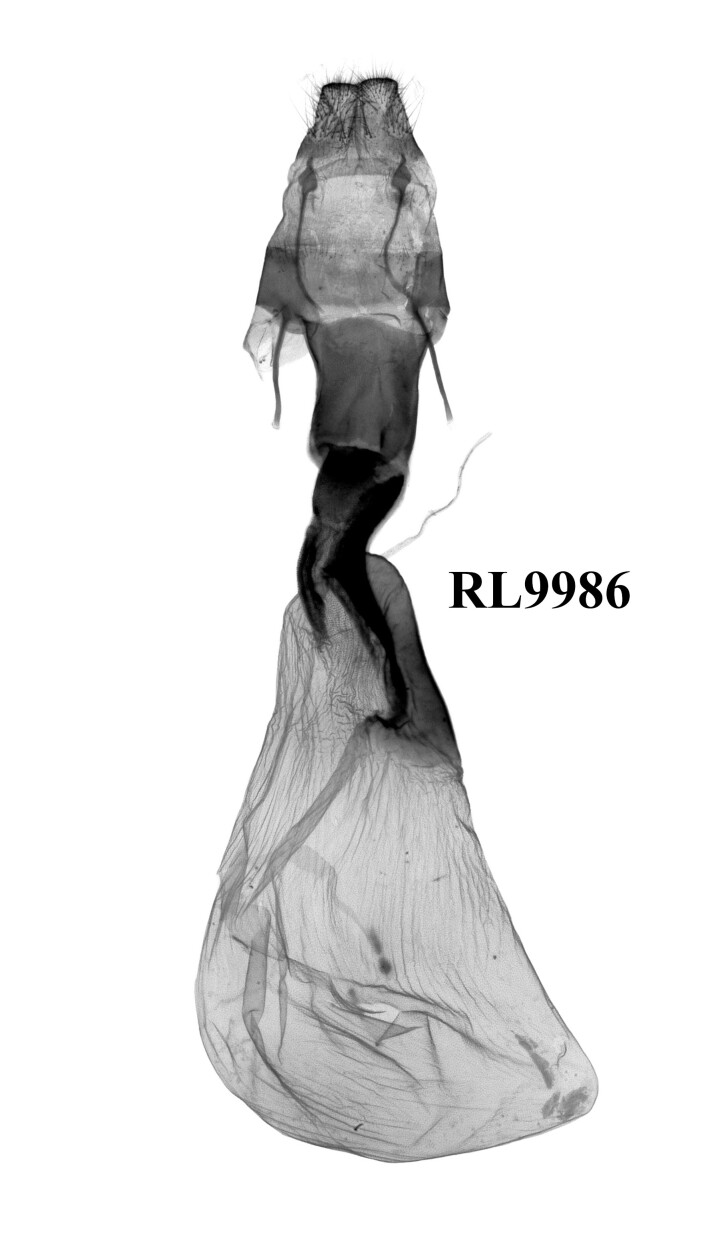
Female genitalia of *Gaurenoglaeaalternata*, paratype, W. Sichuan, China (slide RL9986f).

**Figure 6.**
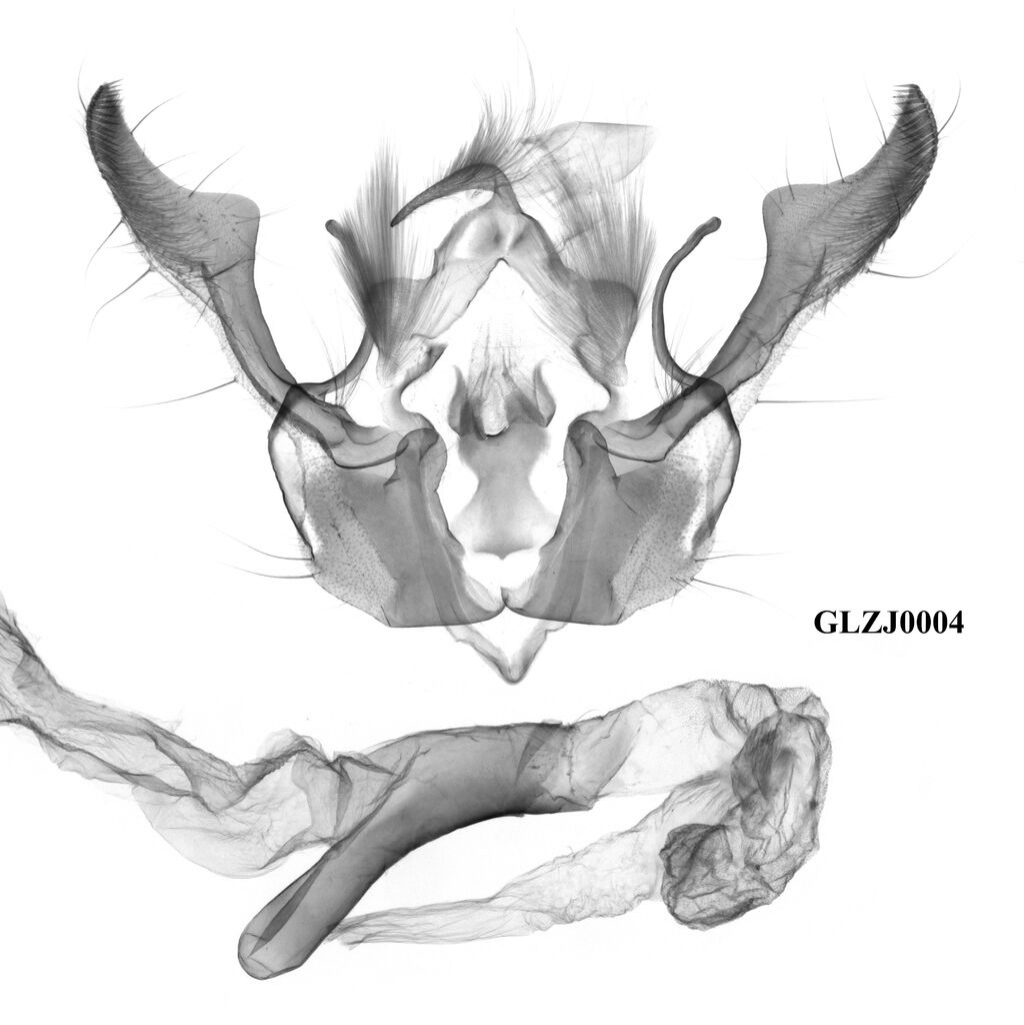
Male genitalia of *Hemiglaeacostalis*, Chunan County, Hangzhou City, Zhejiang Province, China (prep. GLZJ0004m).

The external appearance of the species in this genus is distinctive within the tribe Xylenini (Figs 1, 2, 3). The ground colour of their forewings is dark brown, with numerous large and small ochreous patches distributed, which makes them most similar to *Houlberthosiaornatissima*. However, their wingspan is relatively small, the compound eyes are uncovered and their tegulae are dark brown, lacking an ochreous-white colouration. The colour of their abdomens is also distinct. Moreover, the patches on the forewing of *Gaurenoglaea* species are more ochreous and the hind-wings are brown rather than a glossy milky ochreous colour. Regarding male genitalia (Fig. 4), all three known species of this genus display remarkably similar characteristics: the weak tegumen with well-developed penicular lobes, the large, ribbed-folded clavus and especially the well-developed falcate cucullus and long, arc-shaped harpe, which render them similar to those of *Hemiglaeacostalis* (Butler, 1789) (Fig. 6) and *Hemiglaeajumla* Hreblay & Ronkay, 1999. However, the juxta of *Gaurenoglaea* species is narrower and the aedeagus shows significant differences. Their aedeagus shows a well-developed ventral carinal bar and ventro-lateral carinal bar (the ventro-lateral carinal bar is reduced in *G.medogensis* sp. nov.) and the vesica has nail-shaped or long-spiny terminal cornuti. Based on *G.alternata* (Fig. 5), the most prominent feature of the female genitalia is the drop-shaped and membranous corpus bursae as well as the double sclerotised process that extends into the basal portion of the appendix bursae.

#### Distribution

**Figure 7.**
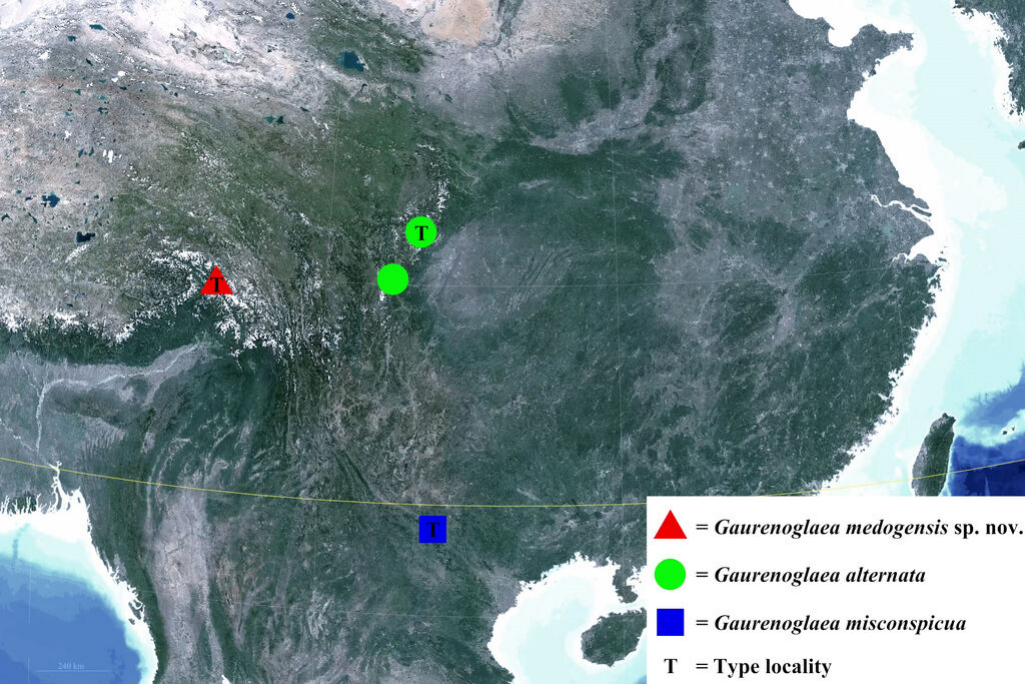
Distribution map of the genus *Gaurenoglaea* in the current study.

Species of this genus are known from Vietnam (Lao Cai) and south-western China (Sichuan and south-eastern Xizang Autonomous Region) (Fig. 7).

#### Biology

The species of this genus are adapted to be active from autumn to winter (from early October to early February) in mountainous areas of medium to high altitudes (2100-2800 m).

#### Species content of *Gaurenoglaea*

*G.misconspicua* Ronkay, Ronkay, Gyulai & Hacker, 2010

*G.alternata* Ronkay, Ronkay, Gyulai & Hacker, 2010

*G.medogensis* **sp. nov.**

### Gaurenoglaea medogensis

Guo & Wang sp. nov.

A1CB6025-FC72-5AF4-A0F3-534EA01512E9

C0B36B9D-68F1-45ED-94FA-AE974D88FB51

#### Materials

- **Type status:** Holotype. **Occurrence:** recordedBy: Liang Guo, Ziqi Yuan & Chuhang Qiao; sex: male; occurrenceID: 5ABF82C9-F081-53A8-A66E-6698DC393023; **Location:** country: China; stateProvince: Xizang Autonomous Region; locality: Linzhi City, Motuo County, Gedang Countryside; verbatimElevation: 2800 m; verbatimLatitude: 29°27′22.04″ N; verbatimLongitude: 95°57′37.24″ E; **Event:** year: 2023; month: 11; day: 11; **Record Level:** institutionCode: SCAU
- **Type status:** Paratype. **Occurrence:** recordedBy: Liang Guo, Ziqi Yuan & Chuhang Qiao; sex: 3 males; occurrenceID: E3173BAE-F228-568B-9A3D-341D2493CCC1; **Location:** country: China; stateProvince: Xizang Autonomous Region; locality: Linzhi City, Motuo County, Gedang Countryside; verbatimElevation: 2800 m; verbatimLatitude: 29°27′22.04″ N; verbatimLongitude: 95°57′37.24″ E; **Event:** year: 2023; month: 11; day: 11; **Record Level:** institutionCode: SCAU

#### Description

**Male** (Fig. 1). Forewing length 17-18 mm (n = 4, 18 mm in holotype), wingspan 36-38 mm (n = 4, 38 mm in holotype). Head densely covered with ochreous and dark brown hairs; labial palpus normal and dark; eyes naked; antenna long and filiform, dark brown in basal and gradually turning brown or reddish-brown at a third of the length from basal to distal end. Thorax mainly dark brown and mixed with some ochreous hairs especially posterior; collar ochreous, tegula dark brown. Forewing narrow, nearly triangular, apically elongated slightly; forewing ground colour dark brown; two basal patches present, the costal one very large and with a very faint brown short line, the other one flat and narrow; an oval patch containing a small brown nuclear dot lies under the discal cell; orbicular stigma smaller than reniform stigma, the orbicular stigma contains a small brown nuclear dot and the reniform stigma contains two small brown dots; a series of postmedial patches extending from costal margin to inner margin form a curved dotted line; three patches lie from the apex to anal angle in the subterminal field, with sizes becoming smaller in turn; in addition to the above patches, there are three or four small patches between basal patch and postmedial line, two smaller patches between postmedial line and apical patch present at costal margin, two small patches between basal patches and postmedial line present at inner margin; cilia of the outer margin chequered with ochreous and dark brown, the ochreous parts round or nearly square, separated from each other, like the prayer beads. Hind-wing ground colour brown, lighter than forewing; cilia of the outer margin ochreous with brownish apical spot or brown. Abdomen brown dorsally, ventral side ochreous with dark brown medial patches.

**Male genitalia** (Fig. 4a, b, c). Uncus curved, short and fine, with sparse hairs. Tegumen weak, small and low, penicular lobes well developed and large, with thick hair. Clavus very large and ribbed-folded. Cucullus well-developed and falcate, with short and weak corona. Harpe arc-shaped, long and fine. Juxta long, broad basally, thin medio-apically. Saccus normal, V-shaped. Aedeagus thin and cylindrical, the ventral-lateral carinal bar well-developed, with a quite long saw-shaped eversible carinal extension, while the ventro-lateral carinal bar reduced, vesica well-developed with basal tube and distal bulb, diverticula present, terminal cornutus well-developed, divided into some long spines.

**Femal.** Unknown.

#### Diagnosis

The new species can be distinguished from the previous *Gaurenoglaea* species by: **a)** The forewing cilia of the outer margin are chequered with ochreous and dark brown in the new species. The ochreous parts are round or nearly square and are separated from each other, like prayer beads. In *G.misconspicua*, the forewing cilia of the outer margin are widely dark brown. The ochreous parts are quite reduced. In *G.alternata*, the forewing cilia of the outer margin are not really chequered. The ochreous parts are smaller, more or less crescent and the ochreous parts join in a wavy line; **b)** The penicular lobes are larger than both *G.misconspicua* and *G.alternata*; **c)** The ventral carinal bar is well-developed in the new species, with the longest eversible carinal extension within the known species of the genus and possessing the longest spines. The ventro-lateral carinal bar is reduced in the new species, while it is noticeable, large, rasper-like in the other two species.

#### Etymology

The specific epithet *medogensis* is derived from the Tibetan name of its type locality, Medog County.

#### Distribution

Motuo County, Linzhi City, south-eastern Xizang Autonomous Region, south-western China (Fig. 7).

#### Biology

**Figure 8.**
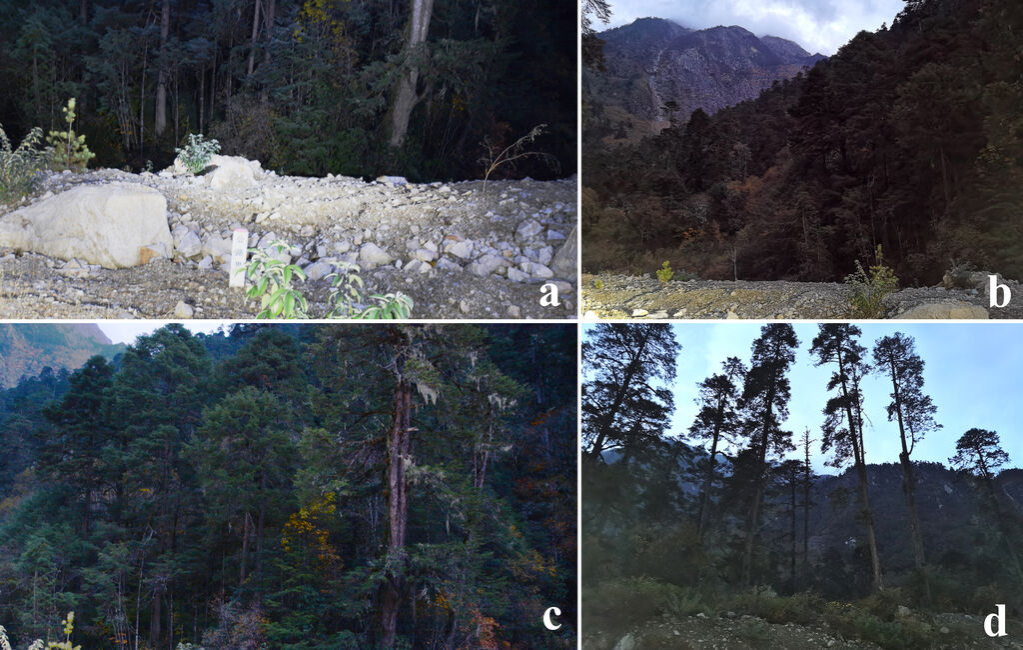
Habitat of *Gaurenoglaeamedogensis* sp. nov., altitude 2800 m, Gedang Countryside, Motuo County, Linzhi City, Xizang Autonomous Region, China.

Four adults were collected on 11-11-2023 at 2800 m altitude during a quite cold night, identifying that the adults are able to endure cold temperatures and fly in late autumn or early winter. In addition, they were collected near the mixed forest, with the coniferous tree being the main component (Fig. 8).

#### Notes

Some evidence indicates that *G.medogensis* and *G.alternata* might exhibit sympatric distribution in Sichuan.

### Gaurenoglaea misconspicua

Ronkay, Ronkay, Gyulai & Hacker, 2010

F38404B1-A01B-56CF-927F-C186E72ECD73

#### Materials

- **Type status:** Holotype. **Occurrence:** recordedBy: Peregovits L. & Ronkay G.; sex: male; occurrenceID: A818E36B-1961-524C-B96C-B48F5FA41E12; **Taxon:** namePublishedIn: Ronkay G., Ronkay L., Gyulai P., Hacker H. 2010. *Esperiana*, 15: 280. fig. 65 (male genitalia), pl. 53, fig. 2 (adult).; **Location:** country: Vietnam; stateProvince: Lao Cai; locality: Fan-si-pan Mts, 7km W Sa Pa; verbatimElevation: 2650 m; verbatimLatitude: 22°18′ N; verbatimLongitude: 103°48′ E; **Event:** year: 1999; month: 2; day: 1-2; **Record Level:** institutionCode: GRB

#### Diagnosis

*G.misconspicua* (Fig. 2) has the typical appearance of this genus. The dark brown parts of the forewing cilia on the outer margin of this species are the most developed within this genus, making it relatively easy to be distinguished. Additionally, regarding the male genitalia (Fig. 4d), the harpe of *G.misconspicua* is the shortest and the juxta is relatively the broadest. Uniquely, the coecum of the aedeagus is characteristically bone-shaped, while it is regularly bulb-like in the other two species.

#### Distribution

This species is only known from Lao Cai, Vietnam (Fig. 7).

#### Notes

The collection time information of the type specimen given in the original literature does not match the label.

### Gaurenoglaea alternata

8404555C-E281-5A71-AFA6-CA4117379F4F

#### Materials

- **Type status:** Holotype. **Occurrence:** recordedBy: local collecter; sex: male; occurrenceID: D1FEFD17-B5EF-5828-B38D-058E84300C9F; **Taxon:** namePublishedIn: Ronkay G., Ronkay L., Gyulai P., Hacker H. 2010. *Esperiana*, 15: 281. fig. 66 (male genitalia), pl. 53, fig. 3 (adult); **Location:** country: China; stateProvince: Sichuan; locality: Volong Reserve, Siguliang Shan; verbatimLatitude: 31°09′ N; verbatimLongitude: 103°20′ E; **Event:** year: 2004; month: 12; day: 1-31; **Record Level:** institutionCode: PGM
- **Type status:** Paratype. **Occurrence:** recordedBy: Floriani & Saldaitis; sex: 2males, 1female; occurrenceID: EFBBEA4E-3E1E-54A0-9512-B7DCE1661D09; **Taxon:** namePublishedIn: Ronkay G., Ronkay L., Gyulai P., Hacker H. 2010. *Esperiana*, 15: 281. fig. 66 (genitalia), pl. 53, fig. 3 (adult); **Location:** country: China; stateProvince: Sichuan; locality: road Ya'an-Kangding, Erlang Shan Mt.; verbatimElevation: 2100 m; verbatimLatitude: 29°51′ N; verbatimLongitude: 102°18′ E; **Event:** year: 2009; month: 10; day: 11; **Record Level:** institutionCode: HNHM
- **Type status:** Other material. **Occurrence:** sex: 2 males; occurrenceID: 14A326CF-08D4-50CE-9456-ABC15D026A74; **Location:** country: China; stateProvince: Sichuan; **Record Level:** institutionCode: PGM

#### Diagnosis

This species (Fig. 3) is similar in appearance to two other *Gaurenoglaea* species. It can be identified by the forewing cilia of the outer margin. The cilia are not truly chequered, the ochreous parts of the cilia are crescent-shaped and they connect in a wavy line. Regarding the aedeagus (Fig. 4e, f), the ventral carinal bar is more developed than *G.misconspicua*, but inferior to *G.medogensis*, while the ventro-lateral plate is the most developed.

#### Distribution

This species is known from two localities in west of Sichuan (Mount Siguniang and Mount Erlang), China (Fig. 7).

#### Notes

There is some evidence suggesting that some specimens produced in Sichuan that were once identified as *G.alternata* are actually *G.medogensis* and we need more specimens from Sichuan to confirm this.

## Identification Keys

### Key to species of *Gaurenoglaea*

**Table:** 

1	The forewing cilia of the outer margin are chequered with ochreous and dark brown, the ochreous parts round or nearly square, separated from each other; the ventro-lateral carinal bar of the aedeagus is reduced.	*G . medogensis* **sp. nov.**
–	The forewing cilia of the outer margin are not chequered or not truly chequered; the ventro-lateral carinal bar of the aedeagus is well- developed.	2
2	The forewing cilia of the outer margin are dark brown; the coecum of the aedeagus is characteristically bone-shaped.	*G.misconspicua*
–	The forewing cilia of the outer margin are not truly chequered, the ochreous parts of the cilia are crescent-shaped and they connect in a wavy line; the coecum of the aedeagus is regularly bulb-like.	*G.alternata*

## Supplementary Material

XML Treatment for Gaurenoglaea

XML Treatment for Gaurenoglaea medogensis

XML Treatment for Gaurenoglaea misconspicua

XML Treatment for Gaurenoglaea alternata

## Figures and Tables

**Figure 1a.**
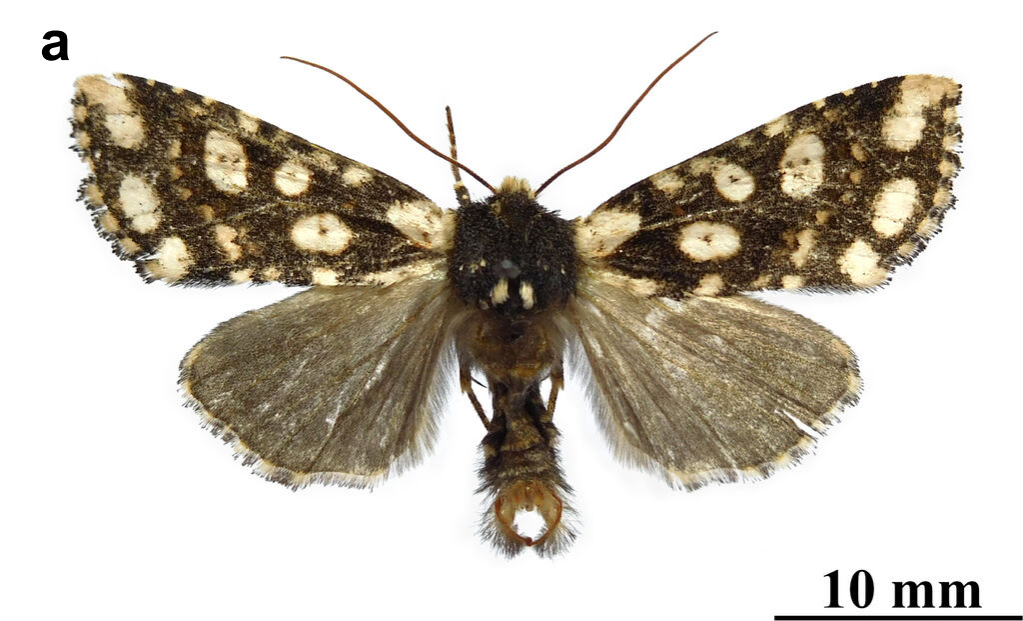
male, holotype, Xizang Autonomous Region, China (coll. SCAU);

**Figure 1b.**
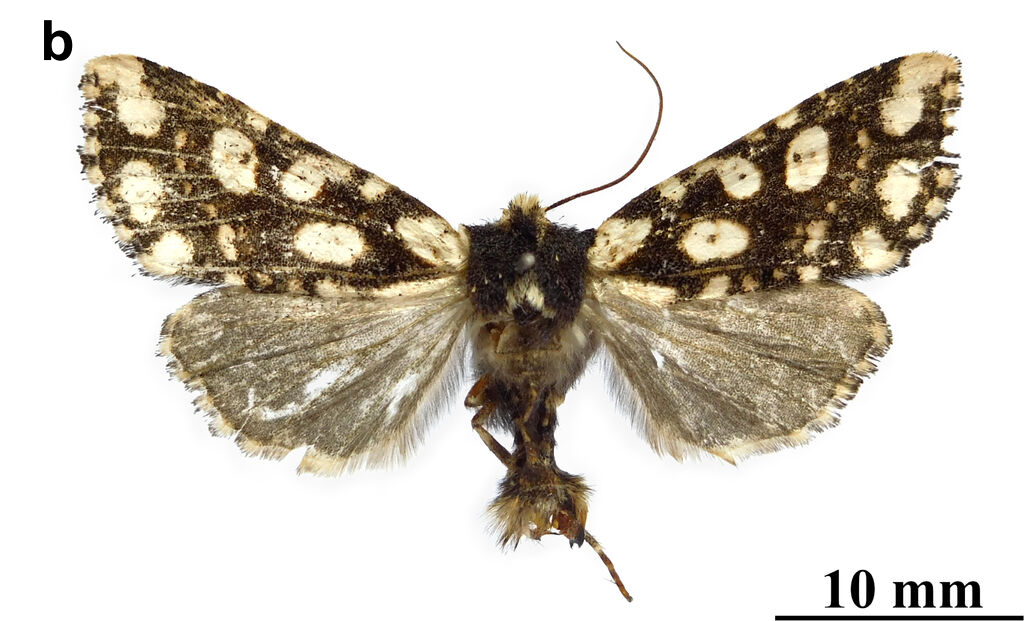
male, paratype, Xizang Autonomous Region, China (coll. SCAU);

**Figure 1c.**
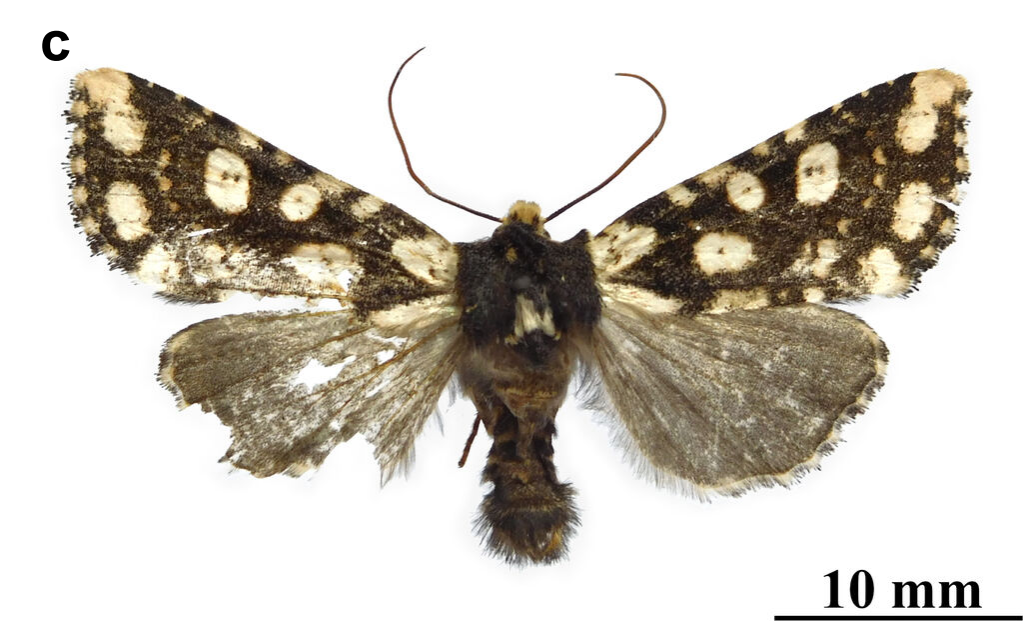
ditto.

**Figure 3a.**
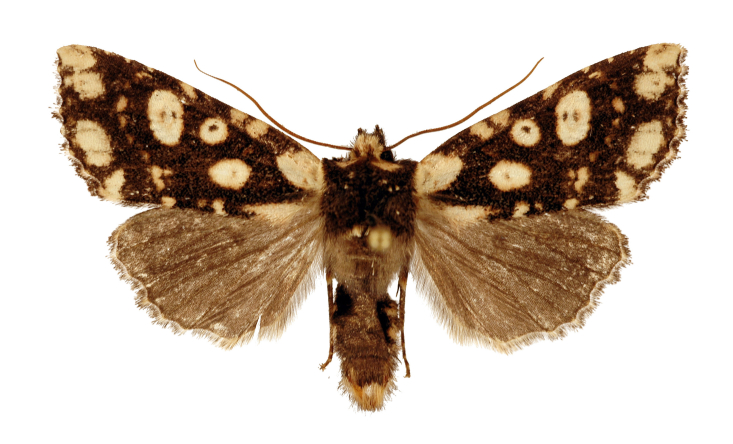
male, paratype, W. Sichuan, China (coll. HNHM);

**Figure 3b.**
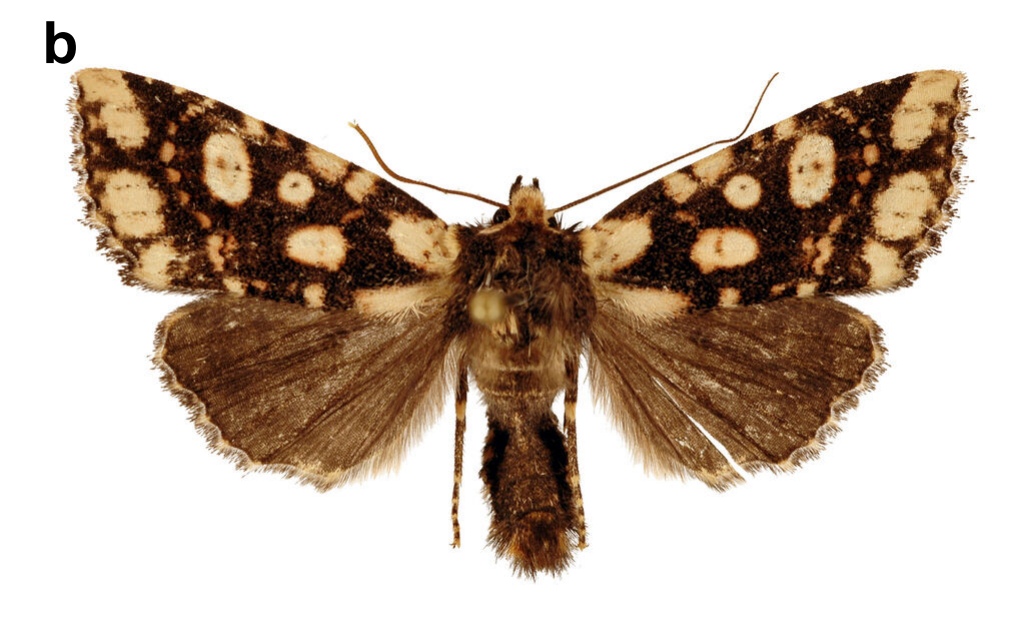


**Figure 3c.**
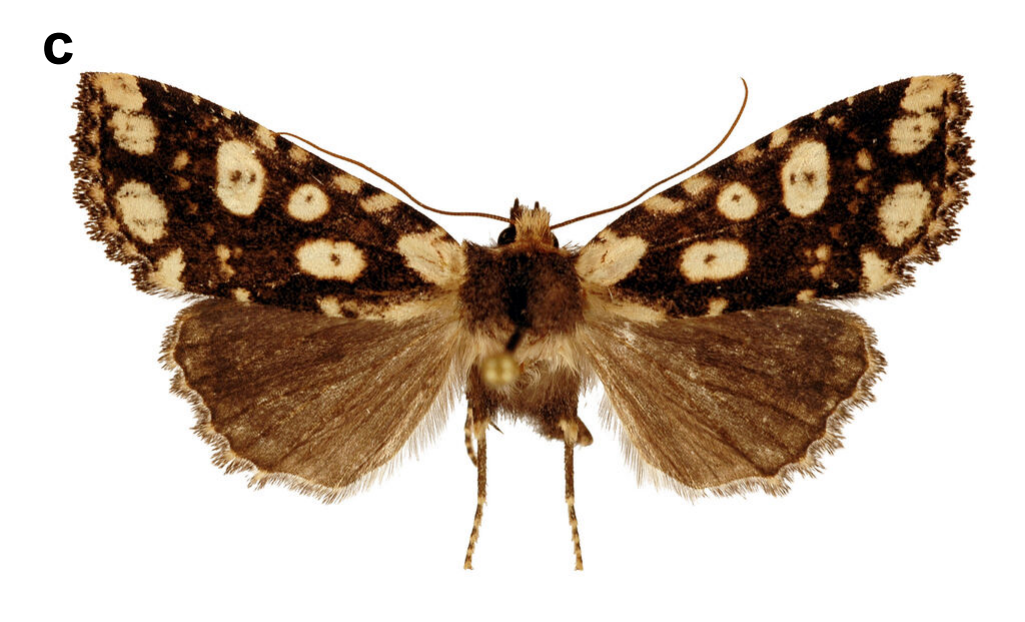
female, paratype, W. Sichuan, China (coll. HNHM).

**Figure 4a.**
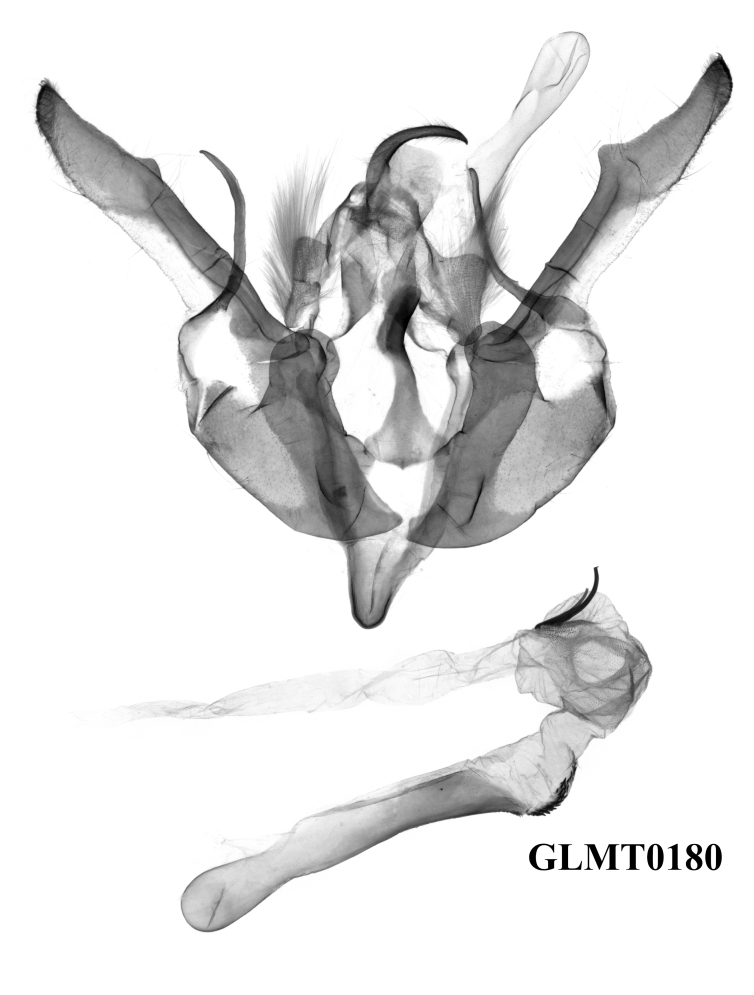
*G.medogensis* sp. nov., paratype, Xizang Autonomous Region, China (prep. GLMT0180m);

**Figure 4b.**
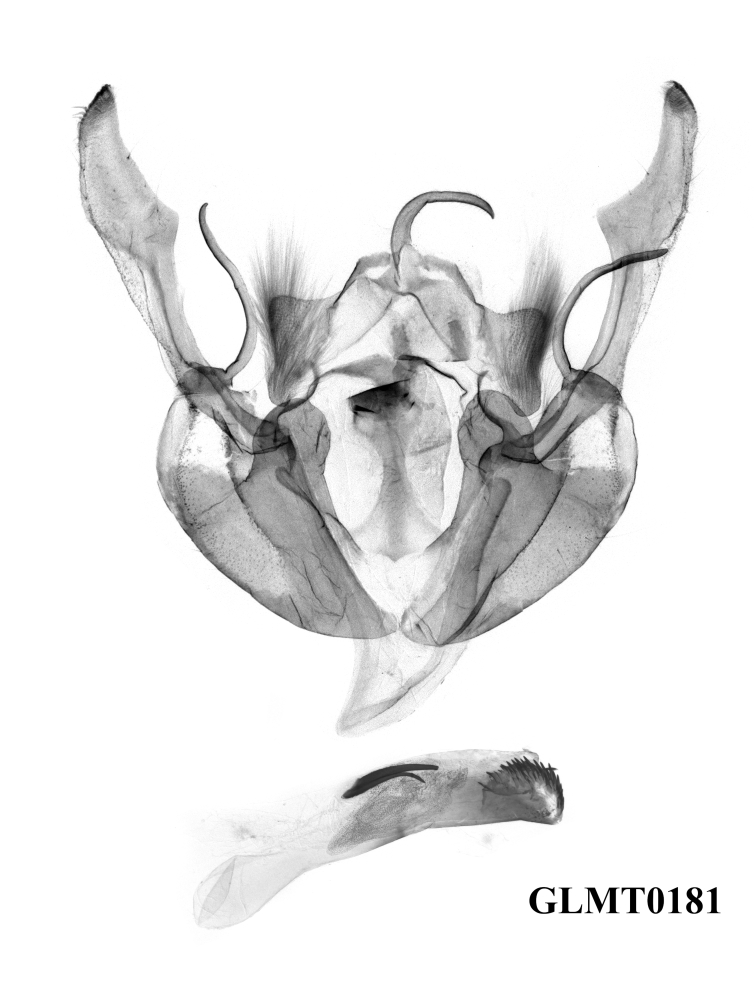
*G.medogensis* sp. nov., paratype, Xizang Autonomous Region, China (prep. GLMT0181m);

**Figure 4c.**
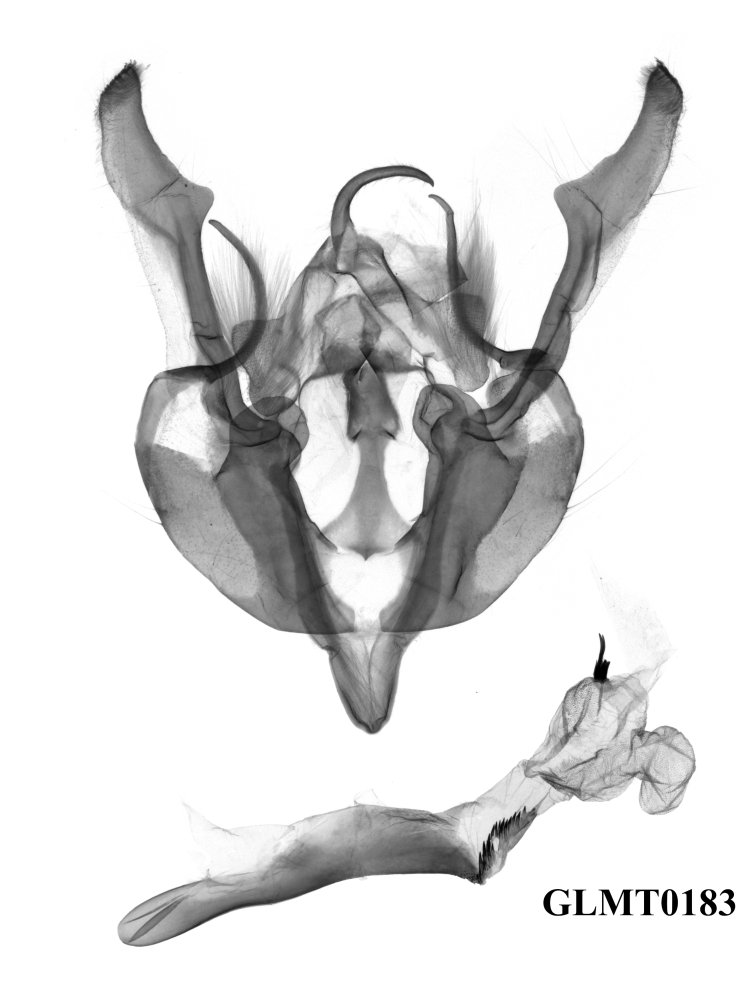
*G.medogensis* sp. nov., paratype, Xizang Autonomous Region, China (prep. GLMT0183m);

**Figure 4d.**
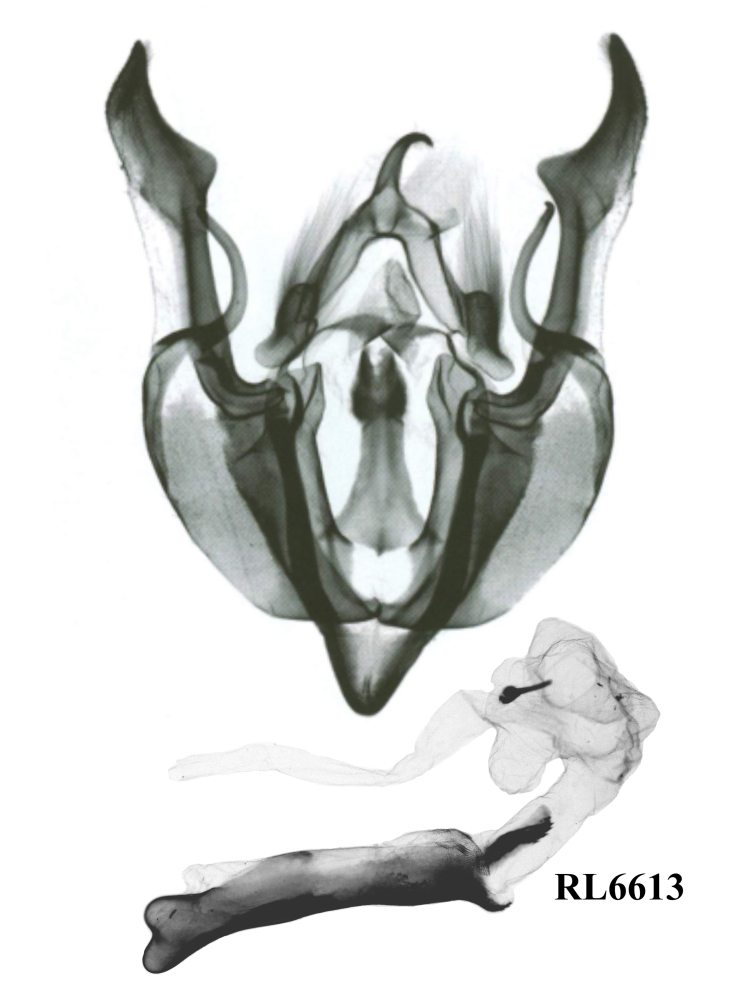
*G.misconspicua*, holotype, Lao Cai, Vietnam (slide RL6613m);

**Figure 4e.**
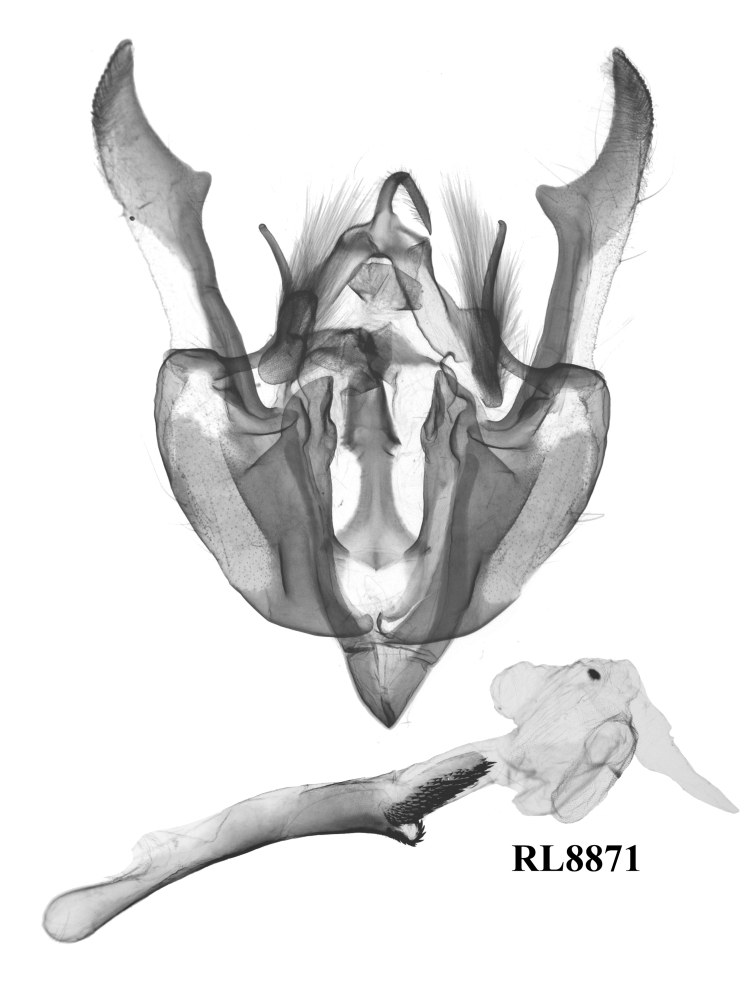
*G.alternata*, holotype, W. Sichuan, China (slide RL8871m);

**Figure 4f.**
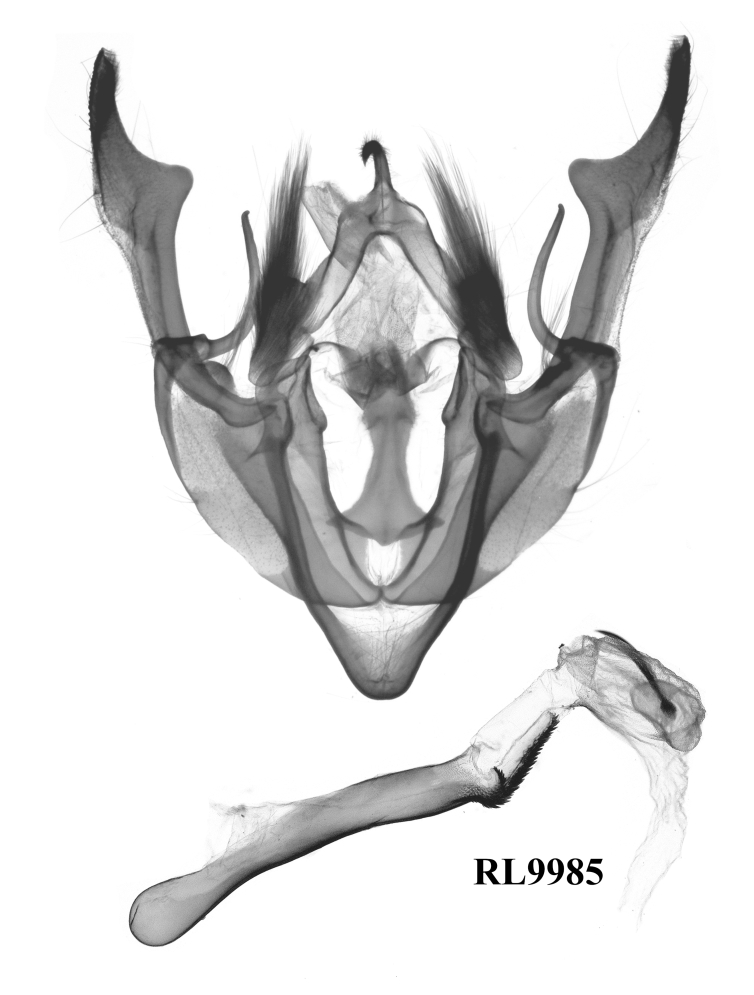
*G.alternata*, paratype, W. Sichuan, China (slide RL9985m).
